# Using Google Trends to Inform the Population Size Estimation and Spatial Distribution of Gay, Bisexual, and Other Men Who Have Sex With Men: Proof-of-concept Study

**DOI:** 10.2196/27385

**Published:** 2021-11-29

**Authors:** Kiffer G Card, Nathan J Lachowsky, Robert S Hogg

**Affiliations:** 1 Faculty of Health Sciences, Simon Fraser University Burnaby, BC Canada; 2 School of Public Health and Social Policy, Faculty of Human and Social Development, University of Victoria Victoria, BC Canada

**Keywords:** gay, bisexual, and other men who have sex with men, spatial distribution, population size estimation, pornography, technology-aided surveillance

## Abstract

**Background:**

We must triangulate data sources to understand best the spatial distribution and population size of marginalized populations to empower public health leaders to address population-specific needs. Existing population size estimation techniques are difficult and limited.

**Objective:**

We sought to identify a passive surveillance strategy that utilizes internet and social media to enhance, validate, and triangulate population size estimates of gay, bisexual, and other men who have sex with men (gbMSM).

**Methods:**

We explored the Google Trends platform to approximate an estimate of the spatial heterogeneity of the population distribution of gbMSM. This was done by comparing the prevalence of the search term “gay porn” with that of the search term “porn.”

**Results:**

Our results suggested that most cities have a gbMSM population size between 2% and 4% of their total population, with large urban centers having higher estimates relative to rural or suburban areas. This represents nearly a double up of population size estimates compared to that found by other methods, which typically find that between 1% and 2% of the total population are gbMSM. We noted that our method was limited by unequal coverage in internet usage across Canada and differences in the frequency of porn use by gender and sexual orientation.

**Conclusions:**

We argue that Google Trends estimates may provide, for many public health planning purposes, adequate city-level estimates of gbMSM population size in regions with a high prevalence of internet access and for purposes in which a precise or narrow estimate of the population size is not required. Furthermore, the Google Trends platform does so in less than a minute at no cost, making it extremely timely and cost-effective relative to more precise (and complex) estimates. We also discuss future steps for further validation of this approach.

## Introduction

Understanding the spatial distribution and population size of marginalized populations allows public health leaders to advocate for population-specific needs, plan and implement relevant treatment and prevention programs, and evaluate the population impact of interventions tailored to these groups [1]. Multiple data sources are needed to triangulate these estimates if we are to provide useful information to public health practitioners.

Gay, bisexual, and other men who have sex with men (gbMSM) are one population for whom sampling frame data are not available. Thus, population size estimation studies are common for this population. In fact, more than 100 gbMSM population size estimation studies have been conducted globally—each aiming to provide precise, accurate, and region-specific estimates [2]. These studies utilize a range of population size estimation methods (eg, census and enumeration, multiplier, capture-recapture, population survey, network scale-up, wisdom of the crowds) in recognition that any given method relies on difficult-to-meet or unvalidated theoretical assumptions or complex and difficult implementation strategies [1]. Nevertheless, various methods tend to converge around similar estimates in a given region. For example, Rich and colleagues estimated the size of the gbMSM population in Metro Vancouver by using the Canadian Community Health Survey, HIV testing service data, the multiplier method (ie, using prevalence of service use data from a representative population-specific data set and multiplying it by the number of people who used a service), and the “wisdom of the crowds” method (ie, asking people to guess). They found that the median of all estimates represented 2.9% of the Metro Vancouver census male adult population with an interquartile range of 1.1%-4.5% [3].

Regardless of the convergence of these estimates, it is difficult to create estimates that accurately reflect the population size of gbMSM in all regions (especially for subregions such as cities and towns)—even in a country with as robust a research infrastructure as Canada. This is largely due to the lack of census data and the paucity of representative samples that include sexual orientation measures. Therefore, while the spatial distribution of gbMSM is known to be heterogeneous, particularly with regards to rural and urban locations [4-6], it is very difficult to ascertain a population size estimate for many regions in Canada. For example, the Pacific AIDS Network in British Columbia estimated that the population share who were gbMSM was 2.6% (of total male population) provincewide, 5.3% in Vancouver Coastal Health Region (ie, the most urban region), and less than 2% in all other health regions [7]. It is unclear whether these differences arise from survey response rates, differences in service utilization, or some other confounder arising from the varying health needs and greater desire for anonymity among rural gbMSM. Different sources of data are undoubtedly expected to capture different populations, and many methods used (eg, clinic samples) cannot necessarily tie individuals to a specific geographic subregion.

Passive surveillance strategies such as social media or internet-based surveillance programs could help validate existing methods by addressing the issue of sampling bias [8,9]. The movement toward these passive approaches may make the process of sample size estimation easier. Such an approach would be consistent with efforts such as Google Flu, which used Google search queries to predict flu outbreaks and hospitalizations [10]. Similarly, the Food Safety project used Twitter data to identify and respond to food poisoning incidents [11]. Clearly, the use of social media and internet search data has public health utility and could therefore be used to help estimate the spatial distribution and population size of gbMSM in cities across Canada.

## Methods

**Figure 1:**
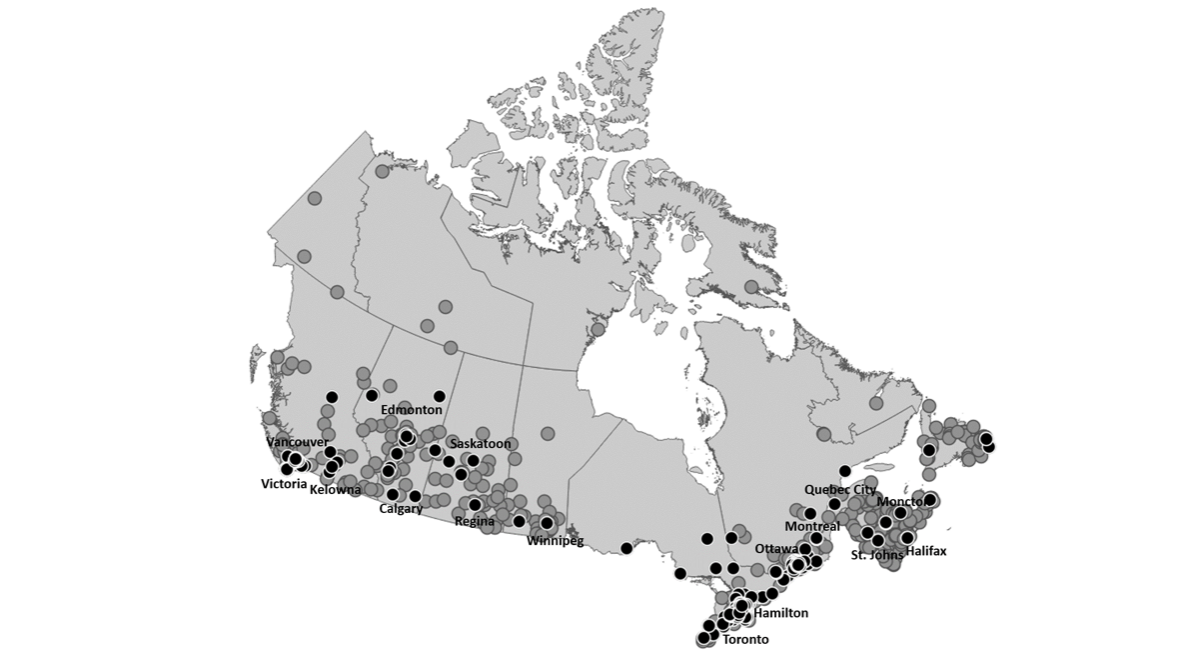
Cities from Google Trends with prevalence estimates for the “gay porn” search term in Canada in 2015-2019. Gray circles represent cities with prevalence estimates >0%, while black circles represent cities with prevalence estimates of +1%.

On October 26, 2020, publicly available Google Trends [12], based on internet search behavior between January 1, 2015 and January 1, 2020, was used to estimate the percentage of searches for “gay porn” (quotation marks not included) relative to the searches for “porn.” The resultant data provided the relative proportion of searches for each given term. The data therefore control for population. Google Trends data also omit outliers (ie, individuals searching for the same term in a short period of time) [13]. These values were thus interpreted as the prevalence of gay porn searches, which we propose as a simply proxy indicator as a proof of concept for how Google Trends data might be leveraged to estimate the distribution of gbMSM. We acknowledge, of course, that only men do not search for porn and that the frequency of porn searches may vary geographically and between sexual orientations. Given that men and gbMSM, in particular, may be more likely to consume porn and that straight men and women may also search for gay porn, we believe this indicator should likely be interpreted as an overestimation of the prevalence of gbMSM in any given area [14,15]. Furthermore, gbMSM living in rural areas may be more likely to consume pornography (perhaps due to reduced access to sexual partners [16]). With consideration of the indicator’s limitations, subregion breakdowns for available cities were extracted, resulting in prevalence estimates of the “gay porn” search term for 609 cities (See Figure 1). Coordinates for each city were retrieved using the Google Geocoding application programming interface [17], and a cartographic file was constructed with latitude, longitude, and our estimate of the prevalence of the “gay porn” search term. Additional cartographic files for census subdivision and provincial boundaries were also accessed via Statistics Canada [18].

All data files were uploaded into QGIS and projected to the NAD83 Statistics Canada Lambert Conformal Conic projection (EPSG 3347). Using the inverse distance weighting (IDW) interpolation tool, a raster was created. Standard settings were used for the IDW interpolation. The distance coefficient (p) was set to 2.0. in order to provide a map of the areas with the highest prevalence of the “gay porn” search term and the pixel size was set to 1000. Owing to overdispersion from a high number of 0 values, these values were omitted from the data set upon which IDW values were based under the assumption that the mechanisms producing 0% estimates are likely attributable to poor quality data from low search volume regions. As such, IDW-interpolated spatial distributions were calculated from data on 134 cities (21.9% of all 611 Canadian cities tracked by Google Trends).

In addition to the nationwide figure, subregional figures for selected Canadian cities were also captured and displayed at a 1:1210020 scale. Regression models were also constructed to examine the association between the prevalence of the “gay porn” search term and population density. Population density estimates were taken from the 2018 Canadian census for each of the 609 cities included. City-level data (noninterpolated) were used for the regression analysis, and the cities with 0 values were included. Moran’s I was calculated to assess for spatial autocorrelation between point estimates of the prevalence of the “gay porn” search term. Based on these results, we constructed a linear mixed-effects regression model with linear, exponential, Gaussian, and spherical spatial correlation structures. Minimal differences between the models were observed, but we selected the model with a spherical correlation structure that had the lowest Akaike information criterion value. As all data for this analyses were publicly available, this study did not require review by a research ethics board.

## Results

**Figure 2:**
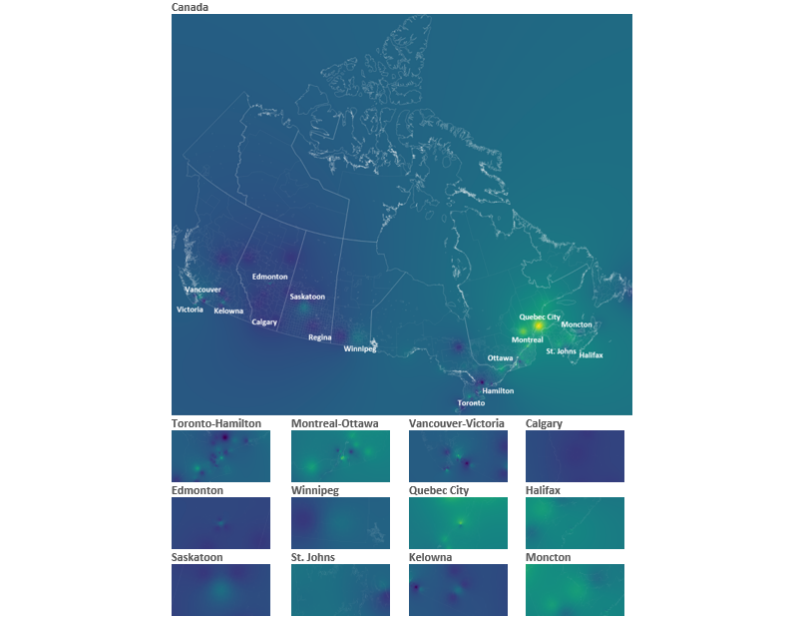
Inverse distance weighted prevalence of the “gay porn” search term in Canada in 2015-2019. Yellow/green estimates higher prevalence, while darker shades of blue represent lower prevalence.

**Figure 3:**
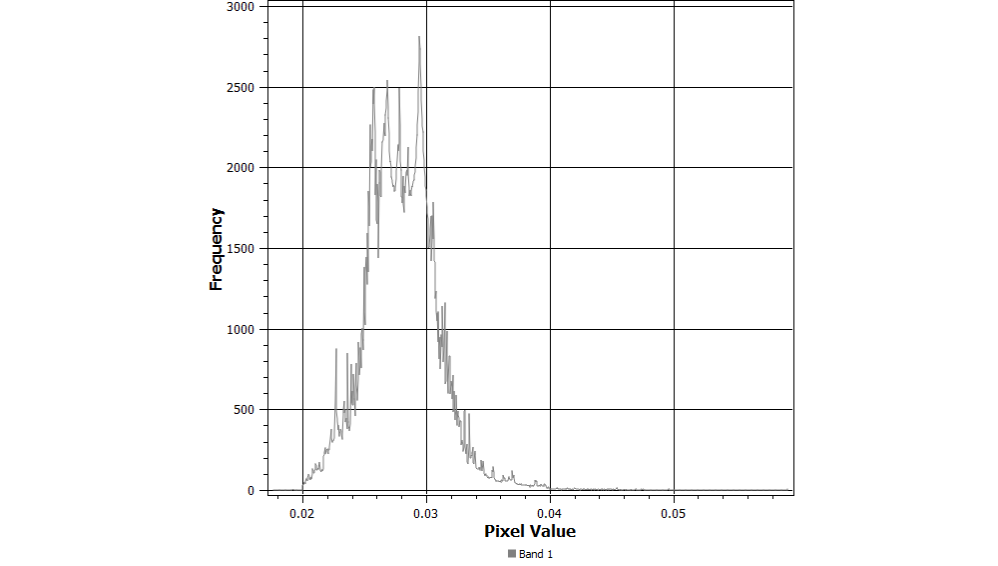
Raster histogram of interpolated prevalence of the “gay porn” search term in Canada in 2015-2019.

The mean prevalence of the “gay porn” search term for all cities retrieved from Google Trends was 0.6% (SD 0.124). If the 0% prevalence estimates are removed, the estimated prevalence increases to 2.8% (SD 0.008). Data were right skewed, with only 6 Canadian cities having prevalence estimates of 5% or higher (ie, Vancouver, Rimouski, Saguenay, Côte Saint-Luc, Montreal, and Quebec). Several cities had estimates of 4% (Toronto, Ottawa, New Westminster, Victoria, Burnaby, Moncton, Dieppe, Fredericton, Halifax, Stratford, Sept-Iles, Mascouche, Gatineau, Drummondville, Longueuil, Sherbrook, and Brossard). Based on Moran’s I, the prevalence of the “gay porn” search term was spatially autocorrelated (observed=0.156, expected=–0.002, SD 0.008, *P*<.001). Regression results adjusted for the spatial correlation structure showed that higher population density was statistically related to the prevalence of the “gay porn” search term: each 100-person increase in population density was associated with a 0.07% increase in the prevalence of the “gay porn” search term (β=.00074, SE 0.000062, *P*<.001). The interpolated prevalence of the “gay porn” search term is provided in Figure 2. A histogram of the raster values generated from the IDW interpolation is provided in Figure 3. These results highlight the bulk of estimates to be between 2% and 4%, with a long right tail reflecting values approaching 6%.

## Discussion

### Principal Findings

This proof-of-concept study explored the use of internet-porn search trends, tracked using the free, easy-to-use Google Trends platform, and approximated an estimate of the spatial heterogeneity of the spatial distribution of gbMSM. In doing so, we showed that a somewhat reasonable description of the spatial distribution and population size estimation could be achieved with further development and validation of this estimate. We hypothesized that Google Trends search volume for “porn” and “gay porn” would provide a *good enough* estimate for the distribution of gbMSM across Canada, with estimates available at the city level. Results from our analyses are largely consistent with other estimates of the population size of gbMSM in Canada—falling in the 1%-5% range (with the most probable estimates being somewhere in the 2%-4% range) [6,7]. Our results also highlight the gradient in the prevalence of “gay porn” searches: estimates in the 2% range for more rural locations and the 4%-5% range for large urban population centers such as Vancouver, Toronto, and Montreal. This again is consistent with what is generally understood about the spatial distribution for gbMSM. For example, using much more time-intensive approaches to examine the spatial distribution of gbMSM, Algarin and colleagues [4] and Card and colleagues [5] used geosocial sexual networking apps to find that the higher density of users is strongly correlated with population density. Card and colleagues [5] reported a 3% increase in the number of users for each 100-person increase in population density; Algarin and colleagues [4] reported a 1%-2% increase in the number of users for each 100-person increase in population density (depending on the time of day). These estimates are close to, perhaps a bit higher, compared with our estimate found here, showing a 0.7% increase per 100-person increase in population density. Our underestimation of this relationship relative to previous studies may reflect the ability of Google Trends data to surveil pornography consumption patterns of individuals who might not be identified using alternative methods (eg, hard-to-identify gbMSM). As such, based on passive surveillance approaches—such as those utilized in these examples—it is strongly suggested that denser population centers have a greater proportion of gbMSM relative to the overall population. It remains unclear whether the increase in population density is closer to 3% per 100 persons or 0.7% per 100 persons. Clearly, uncertainty remains in these estimates and population size estimation studies should at least recognize these regional differences and attempt to correct for them in their estimates when the target geography covers a mix of urban and rural areas. Sensitivity analyses using estimates identified in this study and others could provide a range of plausible values for public health professionals to work with.

The strength of using internet search data to estimate the spatial distribution of gbMSM is that it takes less than a minute to obtain a potentially reasonable estimate for a given city, provided of course that the data are available for a given city via Google Trends. However, the feasibility of using internet search data to estimate the spatial distribution of gbMSM is based on several primary assumptions, which are at least partially violated in reality. First, we assume that there is not a confounding effect arising from differences in the prevalence of porn consumption between gbMSM and other internet users. In reality, we know that sexual minority men use pornography earlier and with greater frequency compared to heterosexual people, and men use it more than women [19,20]. For example, a study of Danish adults aged 18-30 years showed that 26.2% of men watched porn in the last 24 hours compared to 3.1% of women and that only 6.9% of women watched porn 3 times per week or more compared to 38.8% of men [14]. Similarly, by sexual orientation, heterosexual men (29.5%) in the United States are less likely to view pornography online once a day or more compared to both gay (51.3%) and bisexual (52.6%) men [15]. Given these statistics, it is possible that our estimates may as much as double the population size estimates. Adjusting for these differences would bring our estimates in line with those of others, which suggest that between 2% and 4% of the males (rather than total population) are nonheterosexuals [6,7]. Second, we assume that searching for gay porn or porn is a sufficient representation of sexual orientation. It should be obvious that in reality, we do not know the gender or sexual orientation of individuals searching for “porn” or “gay porn.” This is underscored by the reality that gay porn use is not limited to gbMSM, and the relationship between these various constructs is dependent on recall period and how sexual orientation is defined [21]. Indeed, sexual orientation can be defined by behavior, attraction, or identity—all three of which can vary over time. Conversely, not all gay men would necessarily use the prefix “gay” when searching for porn. Third, we assume that the prevalence of porn search among gbMSM and non-gbMSM internet users does not vary geographically. In reality, we know that factors such as financial stress (which does vary geographically) impact the prevalence and frequency of pornography use [22]. Another limitation of this study is that Google Trends data only report the prevalence of search terms to the nearest whole percentage. The lack of a decimal point challenges the use of Google Trends search data for generating precise estimates. Of course, the need for precise estimates (as opposed to generating estimates that are “good enough”) varies depending on the purpose and intent of the professionals using these estimates. For example, the method could be used to help policy makers determine whether there is a sufficiently large gbMSM population to justify the creation of subcommunity-specific health services but may not be sufficient to assess year-to-year variation in the rates of sexually transmitted and blood-borne infections. In instances where precise estimates are needed, sensitivity tests and comparisons between multiple methods will also be required [1]. We also note that the location data available in Google Trends might be obscured by the use of virtual private network software, which can be used to change a user’s location. It is possible that servers located in urban centers might increase the prevalence of search terms. Although little is known about virtual private network usage or about its relationship to sexually explicit media consumption, we anticipate that this error would be small. As this pilot study aims to provide a proof of concept, a simple indicator was used. However, future research could seek to develop better and more precise indicators that leverage one or more platforms (eg, combining weighted estimates from Google Trends with other platforms or by considering open public health data or medical prescription data; identifying multiple keywords with strong discriminate validity). For example, it is necessary to validate how accurately the search term “gay porn” correlates with sexual orientation. Although the added complexity of these future approaches may again move this work beyond its utility, the additional research could be used to validate simple measures such as the one we have used. These challenges reflect the broader issues inherent in utilizing platforms such as Google Trends. However, in situations in which imprecise estimations provide sufficient evidence for informing public health efforts, these tools appear to offer some utility. At the very least, these data provide a point by which data can be triangulated from different sources through the use of scan statistic techniques that could compare patterns arising from different methods.

### Future Research

Although this proof-of-concept study showed that Google Trends data can be feasibly and quickly used to derive an estimate of the spatial and population density of gbMSM, it is our opinion that further experiments and analyses for this metric are required to demonstrate that it provides a reasonably accurate and precise proxy for the true spatial distribution of the gbMSM population. To achieve this validation, we suggest that future research should assess the spatial correlation between the estimates reported using this methodology and those from other surveys or data sources. As we have discussed, studies of the gbMSM population distribution can be difficult for myriad reasons. However, by looking at correlations in patterns of response rates for gbMSM-specific surveys or looking at the prevalence of gbMSM populations within key population centers as found in government surveys, it is likely possible that the validation of this proposed approach could readily be completed.

### Conclusion

The Google Trends prevalence of “gay porn” internet searches relative to “porn” internet searches is a passive surveillance indicator that may approximate existing population size estimates of the gbMSM population down to the municipal level. Although lacking precision, it is a “good enough” estimate, especially considering its relatively minimal demands on financial and human resources for regions with high levels of internet access. Matched with existing methods (which are vulnerable to a different assortment of biases), internet porn searches can help triangulate the validity of subregional population size estimates. If keywords can be identified that allow for comparisons between other marginalized populations or communities, it is possible that search terms on Google Trends could allow for other population sizes to be estimated.

## Abbreviations

gbMSM: gay, bisexual, and other men who have sex with men
IDW: inverse distance weighting
